# Dual-Modal Electrical Imaging of Two-Phase Flow—Experimental Evaluation of the State Estimation Approach

**DOI:** 10.3390/s23094462

**Published:** 2023-05-03

**Authors:** M. Ziaul Arif, Aku Seppänen, Ville Kolehmainen, Marko Vauhkonen

**Affiliations:** 1 Department of Technical Physics, University of Eastern Finland, 70211 Kuopio, Finland; 2 Department of Mathematics, University of Jember, Jember 68121, Indonesia

**Keywords:** dual-modal imaging, electrical tomography, electromagnetic flow tomography, state estimation, two-phase flow

## Abstract

Accurate measurement of two-phase flow quantities is essential for managing production in many industries. However, the inherent complexity of two-phase flow often makes estimating these quantities difficult, necessitating the development of reliable techniques for quantifying two-phase flow. In this paper, we investigated the feasibility of using state estimation for dynamic image reconstruction in dual-modal tomography of two-phase oil–water flow. We utilized electromagnetic flow tomography (EMFT) to estimate velocity fields and electrical tomography (ET) to determine phase fraction distributions. In state estimation, the contribution of the velocity field to the temporal evolution of the phase fraction distribution was accounted for by approximating the process with a convection–diffusion model. The extended Kalman filter (EKF) and fixed-interval Kalman smoother (FIKS) were used to reconstruct the temporally evolving velocity and phase fraction distributions, which were further used to estimate the volumetric flow rates of the phases. Experimental results on a laboratory setup showed that the FIKS approach outperformed the conventional stationary reconstructions, with the average relative errors of the volumetric flow rates of oil and water being less than 4%. The FIKS approach also provided feasible uncertainty estimates for the velocity, phase fraction, and volumetric flow rate of the phases, enhancing the reliability of the state estimation approach.

## 1. Introduction

Two-phase flow involving oil and water is commonly encountered in process industries, for example the chemical, petroleum, and food industries [1]. Accurate measurement of the two-phase flow quantities, such as the velocity fields, phase fraction distributions, and volumetric flow rates, plays an important role in managing production and process automation. However, the inherent complexity of the two-phase mixture often causes difficulties in estimating the flow quantities. One possible solution for measuring the flow is the use of a separating technique, which has been implemented in many industries over the years. This technique requires a separator tank to separate the mixture into individual materials and a single-phase flow meter to measure the flow rate of each phase. However, it is an expensive and inefficient method, and due to its invasive nature, it can interrupt continuous industrial processes [2,3]. This calls for developing efficient, reliable, and non-invasive high-speed techniques for quantifying two-phase flow.

In recent years, a considerable number of non-invasive techniques have been introduced for estimating two-phase flow quantities. Tomographic imaging, among those techniques, has evolved into a novel class of techniques for rapid imaging of the distribution of two-phase flow quantities [1]. Electrical resistance tomography (ERT) [4,5], electrical capacitance tomography (ECT) [6,7], electrical impedance tomography (EIT) [8], magnetic induction tomography (MIT) [9], and microwave tomography (MWT) [10,11] are the family of tomography techniques that are commonly used for estimating phase fractions and for characterizing flow patterns in two-phase flow. Nevertheless, a single-modal tomography is usually unable to retrieve sufficient information on the process quantities, especially the volumetric flow rates of the phases, as this would require simultaneous reconstruction of more than one spatio-temporally distributed quantity, namely the phase fraction distributions and the velocity field. Therefore, dual-modal tomography techniques have become more prevalent and attractive recently and are being currently developed widely for quantifying two-phase flow because of their capability to retrieve two distinct quantities [2].

Several dual-modal tomography systems have been proposed and recently implemented in two-phase flow measurement, such as ECT-ERT [12], inductive flow tomography-ERT [13], magnetic induction tomography and electromagnetic velocity tomography [14], ECT-GRT [15], and electromagnetic flow tomography and electrical tomography [16]. Often, as in the above cited works, the image reconstructions of the two tomographic modalities are performed separately, even though the systems have interconnected parameters. In some studies, image reconstruction has been carried out using so-called fusion methods to improve the reconstructions [17]. For example, the image-based fusion method, which combines images reconstructed separately by each system, has been applied for imaging oil–gas two-phase flow using dual-modal ECT and Gamma-ray tomography (GRT) [17,18]. Furthermore, the articles [18,19] demonstrated that the use of a data fusion method can improve the reconstruction quality in dual-modal tomography. However, these methods typically require post-processing of the data, which can be time-consuming and may not be suitable for real-time applications.

In recent work, a joint reconstruction approach has been proposed for the dual-modal tomography system involving electromagnetic flow tomography (EMFT) and electrical tomography (ET) [20]. In this approach, the contribution of the conductivity distribution to EMFT measurements was modeled when computing the EMFT-based velocity reconstruction, and statistical correlations of the conductivity distribution and velocity were accounted for in the forms of spatial priors. While the image reconstructions in the dual-modal tomography in [20] were based on the joint reconstruction of the velocity field and the conductivity distribution, the information on the interconnection of these quantities included in the reconstruction was still incomplete. This is because the velocity field of the two-phase fluid governs the temporal evolution of the conductivity/phase fraction distribution, and this information cannot be fully accounted for in the conventional, stationary image reconstruction.

To address this issue, in [21], the dual-modal imaging problem of EMFT and ET was recast in the framework of Bayesian state estimation. In this work, the temporal evolution of the phase fraction under the influence of the velocity field was modeled with a stochastic convection–diffusion (CD) equation, and based on the EMFT and ET observations, the time-dependent velocity field and phase fraction distribution were reconstructed simultaneously using the extended Kalman filter (EKF) and fixed-interval Kalman smoother (FIKS). This approach takes into account the interconnection between the velocity field and the phase fraction distribution and enables the reconstruction of both quantities with improved accuracy compared to conventional methods. In [21], the proposed approach was tested only with synthetic data, showing promising results.

Furthermore, although the CD-model-based state estimation has been validated experimentally for a single-modal tomography (ERT) [22,23,24], its performance for dual-modal tomography with experimental data is not yet established. Therefore, the aim of this paper was to study the performance of the state estimation approach with the CD model when applied to experimental EMFT and ET data. In the set of experiments, two-phase flow of oil–water mixtures with a total oil volume fraction of 10–40% were considered, in cases of various stationary average flow speeds and in a non-stationary case where the flow speed varied during the experiment. The proposed approach was shown to provide more accurate and reliable estimation of two-phase flow quantities, with potential applications in various process industries.

## 2. Methodology

### 2.1. State Estimation in Dual-Modal Imaging

In this section, we review the state estimation approach to EMFT–ET dual-modal imaging proposed in [21]. First, in Section 2.1.1, we review the observation models of EMFT and ET. In Section 2.1.2, the convection–diffusion model used for approximating the evolution of the two-component fluid is reviewed, and finally, in Section 2.1.3, the dual-image reconstruction problem is cast in the framework of Bayesian state estimation.

#### 2.1.1. Observation Model

Consider two-phase fluid flowing in a pipeline. Let Ω be a segment of the pipe, and denote the inflow and outflow boundaries by $\partial \Omega_{in}$ and $\partial \Omega_{out}$ and the wall of the pipe segment by $\partial \Omega_{wall}$. Further, denote the fluid velocity by $\vec{\upsilon }=\vec{\upsilon }\left(x,t\right)$, where $x=\left(x,y,z\right)\in \Omega \subset R^{3}$ is the spatial coordinate and *t* is time. Further, denote the phase fraction of the dispersed fluid (for example, oil dispersed in water) by ϕ=ϕ(x,t) and the electrical conductivity of the mixture by σ=σ(x,t). In this section, we considered EMFT and ET measurements subjected to the fluid within Ω.

##### Electromagnetic flow tomography (EMFT):

Electromagnetic flow tomography (EMFT) has been extensively studied in the case of single- and two-phase flow for the estimation of the velocity field [25,26]. In EMFT, a set of coils placed around the pipeline are used for generating a magnetic field in the pipe. When the electrically conductive fluid flows under the influence of the external magnetic flux density $\vec{B}=B\left(x\right)$, an electric potential distribution u=u(x) is induced. The induced potential is measured using a set of electrodes that are attached on the inner wall of the pipe.

Based on Maxwell’s equations, the potential *u* satisfies Poisson’s equation:

(1) $$\nabla \cdot \sigma \nabla u=-\nabla \cdot \sigma \left(\vec{\upsilon }\times \vec{B}\right),x\in \Omega .$$

Further, we considered the following boundary conditions:

(2a) $$\begin{array}{cc} \vec{\upsilon } & =0,\;\;\;x\in \partial \Omega_{\mathrm{wall}}, \end{array}$$

(2b) $$\begin{array}{cc} \frac{\partial u}{\partial \vec{n}} & =0,\;\;\;x\in \partial \Omega , \end{array}$$

(2c) $$\begin{array}{cc} \vec{B} & =0,\;\;\;x\in \partial \Omega_{\mathrm{in}}\cup \partial \Omega_{\mathrm{out}}, \end{array}$$

where $\vec{n}$ denotes the outward unit normal vector on the boundary. We approximated the solution of the boundary value problem (1)–(2c) using the finite-element method (FEM). Neglecting the possible radial and angular components of the velocity field and, further, assuming the axial component to be constant along the axial direction, the finite-element (FE) approximation results in the form [25]:

(3) $$\begin{array}{cc} U & =H\left(\sigma \right)\upsilon_{z}+e_{\upsilon_{z}}, \end{array}$$

where *U* is a vector consisting of electrode potentials and $e_{\upsilon_{z}}$ is the additive observation noise. Here, σ and $\upsilon_{z}$ are the finite-dimensional counterparts of the conductivity and axial component of the velocity, respectively, and H(σ) is a forward operator defined as

(4) $$\begin{array}{cc} H(\sigma ) & =MA^{-1}D_{z}\left(\sigma \right)P_{2D\to 3D}. \end{array}$$

Here, $M\in R^{N_{\mathrm{EMFT}}\times N_{3D}}$ is a matrix that defines the measurement pattern, and *A* and $D_{z}\left(\sigma \right)$ are matrices resulting from FE approximations of the left- and right-hand sides of Equation (1), respectively; see [25]. In addition, $P_{2D\to 3D}\in R^{N_{3D}\times N_{2D}}$ is a projection operator extending the 2D velocity profile $\upsilon_{z}=\upsilon_{z}\left(x,y\right)$ to the 3D velocity field along the axial direction *z*. Further, $N_{\mathrm{EMFT}}$, $N_{2D}$, and $N_{3D}$ are the numbers of measurements in EMFT, nodes in the 2D plane, and nodes in the 3D domain Ω, respectively.

##### Electrical tomography (ET):

Electrical tomography (ET) has been a popular method for non-invasively imaging the internal structure of pipes and vessels in various applications [27,28]. In this second modality, we considered ET, which uses electrodes attached on the inner surface of the pipe, $\partial \Omega_{wall}$. In ET, one electrode at a time is set to an excitation potential, and the rest of the electrodes are grounded. In Ω, this results in an electric current density, which depends on the conductivity distribution σ=σ(x). Corresponding to each potential excitation, the resulting currents through all the electrodes, $I_{\ell },\;\ell =1,2,\cdots ,L$, are measured [27]. We note that ET is closely related to electrical resistance tomography (ERT), which uses current injections and potential measurements for determining the conductivity distribution. The reason for using ET instead of ERT in two-phase flow imaging is that, unlike ERT, ET works also in cases of (partly) insulating materials.

We modeled the ET measurements with the so-called complete electrode model (CEM) [27]. In this case, Maxwell’s equations reduce to the form:

(5) $$\nabla \cdot \left(\sigma \nabla u\right)=0,\;x\in \Omega ,$$

and the following boundary conditions were set:

(6a) $$\begin{array}{cc} u+z_{\ell }\sigma \frac{\partial u}{\partial \vec{n}} & =U_{\ell },\;x\in e_{\ell },\;\ell =1,2,\cdots ,L, \end{array}$$

(6b) $$\begin{array}{cc} \int_{e_{\ell }}\sigma \frac{\partial u}{\partial \vec{n}}dS & =-I_{\ell },\;\;\ell =1,2,\cdots ,L, \end{array}$$

(6c) $$\begin{array}{cc} \sigma \frac{\partial u}{\partial \vec{n}} & =0,\;\;x\in \partial \Omega \setminus \overset{L}{\underset{\ell =1}{⋃}}e_{\ell }. \end{array}$$

Here, $e_{\ell }$ denotes the surface of the *ℓ*th electrode, and $z_{\ell }$ is the respective contact impedance. In addition to Equations (5)–(6c), to satisfy the charge conservation law and to ensure the uniqueness of the solution, the following constraints are written:

(7) $$\sum_{\ell =1}^{L}I_{\ell }=0,\;and\;\sum_{\ell =1}^{L}U_{\ell }=0.$$

We approximated the solution of the model (5)–(7) using the FEM; this led to an observation model of the form [28]:

(8) $$\begin{array}{c} I=R\left(\sigma \right)+e_{\sigma }, \end{array}$$

where *I* is a vector of measured currents, R(σ) is the forward model of ET, and $e_{\sigma }$ is an additive observation noise.

##### Observation model for dual-modal imaging:

Next, we combined the observation models of EMFT and ET written above for dual-imaging. Although not necessary, we assumed below that the times of EMFT and ET measurements were matched, so that at each time *t* (more specifically, time index that corresponds to a short time interval), the full frame of ET data and EMFT data corresponding to one magnetic excitation was collected.

Since our aim was at quantifying the flow by estimating the flow rates of phases, we rewrote the models in terms of the phase fraction of the dispersed fluid, ϕ(x,t). Denote the conductivities of the continuous and dispersed fluid by $\sigma^{c}$ and $\sigma^{d}$, respectively. Then, the conductivity of the two-component mixture is [29]

(9) $$\begin{array}{c} \sigma \left(x,t\right)=C\left(\phi \left(x,t\right)\right)=\sigma^{c}\frac{1+2\left(\frac{\sigma^{d}-\sigma^{c}}{\sigma^{d}+2\sigma^{c}}\right)\phi \left(x,t\right)}{1-\left(\frac{\sigma^{d}-\sigma^{c}}{\sigma^{d}+2\sigma^{c}}\right)\phi \left(x,t\right)}\in R^{N_{3D}}. \end{array}$$

Using this model, the observation models of EMFT and ET, (3) and (8), can be rewritten as

(10) $$\left\{\begin{array}{cc} I_{t}=R_{t}\left(\phi_{t}\right)+e_{\phi ,t} & \to \mathrm{ET}\\ U_{t}=H_{t}\left(\phi_{t}\right)\upsilon_{z,t}+e_{\upsilon_{z},t} & \;\;\;\;\;\;\to \mathrm{EMFT}, \end{array}\right.$$

where $R_{t}$ and $H_{t}$ are composition operators defined as $R_{t}\left(\phi_{t}\right):=R_{t}\circ C\left(\phi_{t}\right)$ and $H_{t}\left(\phi_{t}\right):=H_{t}\circ C\left(\phi_{t}\right)$. Here, $\phi_{t}={\left(\phi_{t,1},\ldots ,\phi_{t,N_{3D}}\right)}^{T}\in R^{N_{3D}}$ is a vector consisting of parameters in the finite-dimensional approximation for the phase fraction distribution of the dispersed fluid, $\upsilon_{z,t}={\left(\upsilon_{z,t,1},\ldots ,\upsilon_{z,t,N_{2D}}\right)}^{T}\in R^{N_{2D}}$ contains parameters for the velocity field, and *t* is a discrete time index t=1,…,T corresponding to the times of the sequential EMFT and ET measurements.

To shorten the notations, we define a new state variable $\theta_{t}$ consisting of both the phase fraction and velocity parameters: $\theta_{t}=\left[\begin{array}{c} \phi_{t}\\ \upsilon_{z,t} \end{array}\right]$ and rewrote the system of Equation (10) that form the observation model for EMFT- and ET-based dual-modal imaging [20] as

(11) $$y_{t}=h_{t}\left(\theta_{t}\right)+e_{t},$$

where

$$y_{t}=\left[\begin{array}{c} I_{t}\\ U_{t} \end{array}\right],\;h_{t}\left(\theta_{t}\right)=\left[\begin{array}{c} R_{t}\left(\phi_{t}\right)\\ H_{t}\left(\phi_{t}\right)\upsilon_{z,t} \end{array}\right]\;\mathrm{and}e_{t}=\left[\begin{array}{c} e_{\phi ,t}\\ e_{\upsilon_{z},t} \end{array}\right].$$

The observation noise vector $e_{t}$ was assumed to be Gaussian $e_{t}∼N\left(0,\Gamma_{e_{t}}\right)$, where the covariance matrix $\Gamma_{e_{t}}$ is of the form: $\Gamma_{e_{t}}=\left[\begin{array}{cc} \Gamma_{e_{\phi ,t}} & 0\\ 0 & \Gamma_{e_{\upsilon_{z},t}} \end{array}\right]$.

#### 2.1.2. Evolution Model

In this work, we modeled the evolution of the dispersed fluid using a convection–diffusion (CD) model that describes the transport of the fluid due to both convection and diffusion. Although our goal was to reconstruct the phase fraction of a dispersed fluid in two-phase flow, we modeled its evolution using a single-phase flow CD model approximation [22,30]. In addition to the CD model, we used a non-physical first-order Markov model [31] for the velocity field, which assumes that the velocity at each point in space is dependent only on the velocity at the immediately preceding time step. This model is commonly used in many applications as a computationally efficient approximation. Both of these models are equipped with stochastic terms, which are partly due to model uncertainties—such as the unknown phase fraction on the inflow boundary of the pipe segment—and partly to compensate for the inaccuracies of the simplified flow models. In the numerical studies of [21], state estimation using these models was shown to be feasible also in cases where the true evolution of the target is governed by two-phase flow. The same observation was made based on experimental studies in the context of ERT in the papers [22,23]. In the experiments of the present paper, the feasibility of these models was tested with oil–water flows.

The CD equation for a concentration distribution c=c(x,t) is of the form:

(12) $$\frac{\partial c}{\partial t}=\nabla \cdot \kappa \left(x\right)\nabla c-\vec{\upsilon }\cdot \nabla c,$$

where κ(x) is the diffusion coefficient. As the boundary conditions, we considered

(13) $$\begin{array}{cc} c(x,0) & =c_{0}\left(x\right),\;x\in \Omega , \end{array}$$

(14) $$\begin{array}{cc} c(x,t) & =c_{\mathrm{in}}\left(x,t\right),\;x\in \partial \Omega_{\mathrm{in}}, \end{array}$$

(15) $$\begin{array}{cc} \frac{\partial c(x,t)}{\partial \vec{n}} & =0,\;x\in (\partial \Omega \setminus \partial \Omega_{\mathrm{in}}), \end{array}$$

where $c_{0}\left(x\right)$ is the initial concentration distribution, $c_{\mathrm{in}}\left(x,t\right)$ is the time-varying concentration on the input flow boundary $\partial \Omega_{\mathrm{in}}$, and $\vec{n}$ is the boundary outward unit normal. Since the input concentration is primarily unknown in the model, $c_{\mathrm{in}}\left(x,t\right)$ is considered as a stochastic function:

(16) $$c_{\mathrm{in}}\left(x,t\right)={\bar{c}}_{\mathrm{in}}\left(x,t\right)+\eta \left(x,t\right),\;x\in \partial \Omega_{\mathrm{in}},$$

where ${\bar{c}}_{\mathrm{in}}\left(x,t\right)$ is the expected average concentration and η(x,t) is the stochastic term, which models the uncertainties. Applying the FE approximation of the model (12)–(16) and including an additional stochastic term to account for the model inaccuracies lead to a recursive form [32]:

(17) $$c_{t+1}=F_{t}\left(\upsilon_{t}\right)c_{t}+s_{t+1}\left(\upsilon_{t}\right)+w_{t+1}\left(\upsilon_{t}\right),$$

where $F_{t}\left(\upsilon_{t}\right)\in R^{N_{3D}\times N_{3D}}$ is the evolution matrix, $s_{t+1}\left(\upsilon_{t}\right)\in R^{N_{3D}}$ is the source term associated with the system input $c_{\mathrm{in}}\left(x,t\right)$, and $w_{t+1}\left(\upsilon_{t}\right)\in R^{N_{3D}}$ is the state noise process.

Finally, as in [21], we identified the concentration *c* with the phase fraction of the dispersed fluid ϕ, i.e., we modeled the evolution of $\phi_{t}$ using the FE approximation of the CD model:

(18) $$\phi_{t+1}=F_{t}\left(\upsilon_{t}\right)\phi_{t}+s_{t+1}\left(\upsilon_{t}\right)+w_{t+1}\left(\upsilon_{t}\right).$$

In addition to the evolution model of the phase fraction distribution, we wrote a non-physical first-order Markov model to approximate the evolution of the axially constant velocity field $\upsilon_{z}\left(x,y\right)$ similarly as in [31]:

(19) $$\upsilon_{z,t+1}=\upsilon_{z,t}+\xi_{t},$$

where $\xi_{t}$ is a Gaussian random variable with zero mean and covariance $\Gamma_{\xi_{t}}$ that promotes the spatial smoothness of $\upsilon_{z}\left(x,y\right)$.

Finally, we combined Equations (18) and (19) to form the evolution model for $\theta_{t}$, the state variable in the dual-modal imaging:

(20) $$\theta_{t+1}=f_{t}\left(\theta_{t}\right)+\varpi_{t}\left(\theta_{t}\right),$$

where

$$f_{t}\left(\theta_{t}\right)=\left(\begin{array}{c} F_{t}\left(\upsilon_{t}\right)\phi_{t}+s_{t+1}\left(\upsilon_{t}\right)\\ \upsilon_{z,t} \end{array}\right)\mathrm{and}\varpi_{t}\left(\theta_{t}\right)=\left(\begin{array}{c} w_{t+1}\left(\upsilon_{t}\right)\\ \xi_{t} \end{array}\right).$$

In order to account for the fact that the EMFT only provides an estimate for the axially constant velocity field $\upsilon_{z,t}$, we represent $\upsilon_{t}$ as $P_{2D\to 3D}\upsilon_{z,t}\in R^{N_{3D}}$, where $P_{2D\to 3D}$ was defined earlier. Based on the approximations made in [32], the state noise $\varpi_{t}$ is of the Gaussian form $\varpi_{t}∼N\left(0,\Gamma_{\varpi_{t}}\right)$.

#### 2.1.3. State Estimation

The observation model (11) and the evolution model (20) form a so-called state-space representation, based on which the state variable $\theta_{t}$ is estimated given sequential measurements $y_{t}$.

In Bayesian filtering, the posterior density of the state variable $\theta_{t}$ given measurements $y_{1},\cdots ,y_{t}$ is formed: $p(\theta_{t}|y_{1},\cdots ,y_{t})$. In this work, we approximated the posterior using the extended Kalman filter (EKF), which is based on local linearizations of the models. In EKF, we write $p\left(\theta_{t}|y_{1},\cdots ,y_{t}\right)\approx N\left(\theta_{t|t},\Gamma_{t|t}\right)$, where $\theta_{t|t}$ is the conditional expectation of $\theta_{t}$, i.e., $\theta_{t|t}=E\left\{\theta_{t}|y_{1},\cdots ,y_{t}\right\}$ and $\Gamma_{t|t}$ is its conditional covariance. For the state-space model formed by Equations (11) and (20), the EKF leads to the following recursion [33]:

(21) $$\begin{array}{cc} \theta_{t|t-1} & =f_{t-1}\left(\theta_{t-1|t-1}\right) \end{array}$$

(22) $$\begin{array}{cc} \Gamma_{t|t-1} & =J_{f_{t-1}}\Gamma_{t-1|t-1}J_{f_{t-1}}^{T}+\Gamma_{\varpi_{t-1}} \end{array}$$

(23) $$\begin{array}{cc} K_{t} & =\Gamma_{t|t-1}J_{h_{t}}^{T}{\left(J_{h_{t}}\Gamma_{t|t-1}J_{h_{t}}^{T}+\Gamma_{e_{t}}\right)}^{-1} \end{array}$$

(24) $$\begin{array}{cc} \Gamma_{t|t} & =\left(I-K_{t}J_{h_{t}}\right)\Gamma_{t|t-1} \end{array}$$

(25) $$\begin{array}{cc} \theta_{t|t} & =\theta_{t|t-1}+K_{t}\left(y_{t}-h_{t}\left(\theta_{t|t-1}\right)\right), \end{array}$$

where $J_{f_{t}}$ and $J_{h_{t}}$ are the Jacobian matrices of the mappings $f_{t}\left(\theta \right)$ and $h_{t}\left(\theta \right)$ evaluated at $\theta_{t}=\theta_{t|t}$ and $\theta_{t}=\theta_{t|t-1}$, respectively [21].

While filtering uses data $y_{1},\cdots ,y_{t}$ for estimating $\theta_{t}$ at time *t*, in *Bayesian smoothing*, also the future data are used. Here, we used the so-called fixed-interval Kalman smoother (FIKS), which is applicable for off-line estimation, but we note that the fixed-lag Kalman smoother—which is a nearly on-line estimate—gives practically identical results when the lag (number of future observations) is large enough. In the FIKS, the approximate posterior density $p\left(\theta_{t}|y_{1},\cdots ,y_{T}\right)\approx N\left(\theta_{t|T},\Gamma_{t|T}\right)$ is computed with a backward recursion of the form [33]:

(26) $$\begin{array}{cc} A_{t-1} & =\Gamma_{t-1|t-1}J_{f_{t-1}}^{T}\Gamma_{t|t-1}^{-1} \end{array}$$

(27) $$\begin{array}{cc} \theta_{t-1|T} & =\theta_{t-1|t-1}+A_{t-1}\left(\theta_{t|T}-\theta_{t|t-1}\right) \end{array}$$

(28) $$\begin{array}{cc} \Gamma_{t-1|T} & =\Gamma_{t-1|t-1}+A_{t-1}\left(\Gamma_{t|T}-\Gamma_{t|t-1}\right)A_{t-1}^{T}. \end{array}$$

The calculation of the backward gain matrix $A_{t-1}$ and the estimation error covariances matrix $\Gamma_{t-1|T}$ requires prior calculation of the extended Kalman filter estimates.

#### 2.1.4. Reference Method: Stationary Reconstruction

For reference, we also computed stationary reconstructions for $\phi_{t}$ and $\upsilon_{z,t}$ based on the observation models (3) and (8), using the so-called series reconstruction method as in [20].

The stationary ET and EMFT reconstructions were the Bayesian *maximum a posteriori* (MAP) estimates. They imply the spatial smoothness of the variables $\phi_{t}$ and $\upsilon_{z,t}$ and use the ET-based reconstruction for the conductivity $\sigma_{t}$ in the observation model of EMFT, but do not account for the contribution of the velocity field to the temporal evolution of the conductivity σ or the phase fraction ϕ or the temporal smoothness of the velocity field. For the details of the series reconstruction method, we refer to [20].

#### 2.1.5. Volumetric Flow Rate Estimates

In the experiments of this work, the continuous fluid was water, and the dispersed fluid was oil. The volumetric flow rate of oil ($Q_{t}^{o}$) and water ($Q_{t}^{w}$) at each time step *t* were estimated based on the reconstructed phase fraction distributions and velocity fields using equations:

(29) $$\begin{array}{cc} Q_{t}^{o} & ={\left({\tilde{\phi }}_{t}^{o}\right)}^{T}W\upsilon_{z,t}, \end{array}$$

(30) $$\begin{array}{cc} Q_{t}^{w} & ={\left(1-{\tilde{\phi }}_{t}^{o}\right)}^{T}W\upsilon_{z,t}, \end{array}$$

where ${\tilde{\phi }}_{t}^{o}={\left(\phi_{t,1}^{o},\phi_{t,2}^{o},\ldots ,\phi_{t,N_{2D}}^{o}\right)}^{T}$ and ${\left(1-{\tilde{\phi }}_{t}^{o}\right)}^{T}$ are the reconstructed oil and water fraction distributions, respectively, and $\upsilon_{z,t}$ as given earlier. Furthermore, *W* is a matrix with the elements $W\left(i,j\right)=\int_{A}\varphi_{i}\left(\vec{r}\right)\varphi_{j}\left(\vec{r}\right)dA$, where $\varphi_{i}\left(\vec{r}\right)$ are piecewise linear basis functions used in the finite-element calculations [16].

### 2.2. Experiments

**Figure 1:**
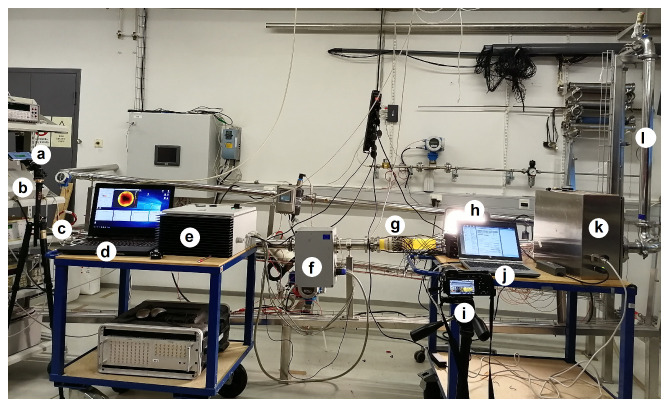
Flow loop installation and dual-modal EMFT and ET system. (**a**) Camera 1; (**b**) ultrasound flow meter device; (**c**) ultrasound flow meter sensors; (**d**) laptop for EMFT; (**e**) signal processor for EMFT; (**f**) EMFT sensors; (**g**) ET sensors; (**h**) back-light; (**i**) Camera 2; (**j**) laptop for ET; (**k**) signal processor for ET; (**l**) vertical pipe segment. In the pipe segment equipped with EMFT and ET sensors, the flow direction is from left to right.

The experiments were carried out in the Electrical Tomography Laboratory at the University of Eastern Finland, Kuopio. The laboratory facilities are shown in Figure 1. In this section, we overview the experimental setup, describe the instruments, and give the details of the test cases.

#### 2.2.1. Flow Loop and Overview of Instrumentation

The total length of the pipeline used in the experiments was 29.90 m; its inner diameter was 0.05 m, and the total volume was about 0.0587 $m^{3}$. It formed a closed loop and had a vertical section (Figure 1l), a horizontal section, and about a $3.5^{\circ }$ inclined section. The liquid was moved in the pipeline using a circulating pump.

The EMFT and ET sensors were mounted in the inclined pipe section in series, as shown in Figure 1f,g. The distance between the EMFT and ET sensors was about 40 cm. This distance was assumed to ensure that no significant interference between EMFT and ET signals occurred. Naturally, this distance also means that the distributions of the velocity and phase fraction were not exactly equal in the positions of the EMFT and ET sensors. However, in the flow conditions considered in this study, the changes in the distributions were relatively smooth, and the approximation of measuring the same target at each time was assumed to be adequate.

For a reference of the flow speed estimation, a commercial ultrasound flow meter was installed in the pipeline (Figure 1b,c). For recording the flow meter data from its display, a video camera (Figure 1a) was used. The approximate spatial average of the velocity given by the ultrasound flow meter is compared to the dual-modal tomography estimates in Section 3.

To visually analyze the flow, a video camera was installed right next to the ET sensor array, as shown in Figure 1i. To avoid problems with interpreting changes in the colors shown by the video, the light conditions and camera settings were kept unchanged during all experiments.

#### 2.2.2. Electromagnetic Flow Tomography System

The EMFT device is shown in Figure 1d–f. A detailed description of the EMFT system used in this work was given in [16,34]. In the EMFT system, four circular coils with an outer radius of 75 mm and 805 copper wire windings were attached to the outer surface of the 150 mm-long pipe segment. For the potential measurements, a ring of 16 circular, stainless steel electrodes (diameter 5 mm) was mounted on the inner surface of the pipe. Axially, the electrode ring was placed in the middle of the pipe segment, i.e., so that the plane spanned by the electrode ring coincided with the central axes of the coils outside the pipeline.

In this study, three excitation patterns were used sequentially, two of them to create nearly uniform magnetic fields along the x- and y-axes and one for a nearly anti-Helmholtz magnetic field. The excitation currents were digitally generated, and the maximum amplitude of the current was set to 1.4 A. During each excitation, the differences between induced potentials in all neighboring electrodes were measured; hence, the EMFT data consisted of 16 potential measurements at each time step. The rate at which all three excitations were run was 3.12 per second. Moreover, to ensure the proper functioning and accurate measurements during the experiments, the EMFT system was calibrated as described in detail in [34].

#### 2.2.3. Electrical Tomography System

The ET system manufactured by Rocsole Ltd., Kuopio, Finland [35,36,37], is shown in Figure 1g,j,k. Two rows of electrodes were mounted on the inner surface of a 120 mm-long pipe segment. Each row consisted of 16 equally spaced stainless steel electrodes.

The ET system excites one electrode at a time to a preset potential with a magnitude of 1.6 V, grounds the other electrodes, and measures the electric currents from the grounded electrodes during each excitation. A total of 496 current measurements were collected at each time step. The frame rate of ET measurements was 9.09 per second.

#### 2.2.4. Test Cases

In the experiments, the two phases of the fluid were: (1) tap water (electrical conductivity of 0.2 $mScm^{-1}$), which was considered as the continuous phase, and (2) cold-pressed rapeseed oil (non-conductive), which was the dispersed phase.

Altogether, 21 tests were carried out; both the phase fraction of oil and the average flow speed were varied between the test cases. Four average volume fractions of oil were considered: 10%, 20%, 30%, and 40%. After adding a suitable amount of oil in each case, the flow loop was run with high speed for some time to make sure that the oil was mixed well enough for the tests. In the case of each total oil volume fraction, dual-modal flow imaging was tested with five average flow speeds: 0.75, 0.90, 1.10, 1.25, and 1.50 ms ${}^{-1}$. In each of these test cases, the average flow speed was set constant for a certain period of time before starting the EMFT and ET data collection, to ensure the stationary average velocity and steady-state of the two-phase flow. All these *stationary average flow speed tests* (Cases 1–20) are listed and named in Table 1.

Finally, we carried out an experiment with a time-varying average velocity in the case of a 40% oil fraction. The average velocity varied over time, starting from about 0.20 ${\mathrm{ms}}^{-1}$, increasing gradually to a maximum average velocity of 1.50 ${\mathrm{ms}}^{-1}$ within about 185 s, holding at 1.50 ${\mathrm{ms}}^{-1}$ for 267 s, and finally, decreasing to about 0.30 ${\mathrm{ms}}^{-1}$ within about 161 s. The resulting flow event with a time-varying velocity field and phase fraction distribution was monitored continuously using EMFT, ET, and the reference sensors described above during the entire period of the experiment. Below, we refer to this *time-varying average flow speed test* as Case 21.

#### 2.2.5. Computational Aspects and Parameter Choices

In the FE approximations of the EMFT and ET forward models, the phase fraction distribution was represented in a piecewise linear basis: In both modalities, the FE mesh consisted of $N_{3D}=2272$ nodes and 9991 tetrahedral elements. The 2D profile of the velocity field was also represented in a piecewise linear basis; the 2D FE mesh consisted of $N_{2D}=581$ nodes and 1096 triangular elements. Further, the electric potential distribution in ET was approximated using a piecewise second-order approximation with an FE mesh consisting of 30,169 nodes and 151,205 elements, as the electric potential densities were found to exhibit large changes in the vicinity of the electrodes. In EMFT, a piecewise linear basis on the FE mesh with 59,632 nodes and 333,263 elements was used [21].

In the FE approximation of the CD-model, the phase fraction distribution was represented in a piecewise linear basis using an FE mesh with 3517 nodes and 18,229 elements. The interpolations between CD and ET/EMFT meshes were performed using linear interpolation.

In the evolution model of the flow speed, Equation (19), different choices for the variance of the stochastic term $\xi_{t}$ were made in the stationary and non-stationary flow cases. The variance of $\sigma_{\xi_{t}}^{2}$ was chosen to be very small between ${\left(1.0\times 10^{-6}\;ms^{-1}\right)}^{2}$ and ${\left(1.0\times 10^{-5}\;ms^{-1}\right)}^{2}$ in Cases 1–20, where the flow speed was not assumed to vary much during the experiment. In Case 21, where the flow speed was varied, the variance of $\sigma_{\xi_{t}}^{2}$ was chosen to be between ${\left(1.0\times 10^{-3}\;ms^{-1}\right)}^{2}$ and ${\left(7.5\times 10^{-3}\;ms^{-1}\right)}^{2}$. Although the choice of this parameter affects the state estimates, based on our previous experience, the reconstructions were not highly sensitive to it [32]. In the observation model, the covariance matrices of the noise variances $e_{\upsilon_{z},t}$ and $e_{\phi ,t}$ were chosen as $(1\%{\left(max\left(\left|U_{t}\right|\right)\right)}^{2}$ [34] and ${\left(0.1\%\left(max\left(I_{t}\right)-min\left(I_{t}\right)\right)\right)}^{2}$ [36,37], respectively.

As noted in Section 2.1.1, the measurements associated with discrete time indices *t* consisted of a full frame of ET data and EMFT data corresponding to one (out of three) excitation. To match the times of the stationary reconstructions with those of the state estimates, the so-called *sliding window method* was used in the stationary reconstructions. That is, to form a full EMFT dataset for each time t≥3, EMFT data from the two preceding time steps t−1 and t−2 were merged with the data corresponding to time *t*.

## 3. Results and Discussion

In this section, the results of the experimental studies are discussed. The results of reconstructing the velocity and oil volume fraction distributions in cases of stationary (Cases 1–20) and non-stationary (Case 21) average flow speeds are considered in Section 3.1 and Section 3.2, respectively. Finally, the estimation of volumetric flow rates is discussed in Section 3.3.

**Figure 2:**
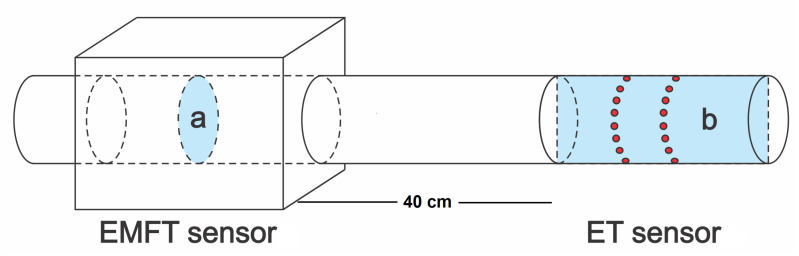
The 2D planes used in the visualization of the estimates. (**a**) Visualization plane of the velocity field; (**b**) visualization plane of oil fraction distribution.

Below, the reconstructions of the velocity field are illustrated on a cross-sectional plane in the middle of the pipe segment covered by the EMFT sensor. The 3D oil fraction distributions are visualized on a vertical plane crossing the central axis of the pipe segment covered by the ET sensor. The geometries of the visualization planes are illustrated in Figure 2.

### 3.1. Cases 1–20: Imaging of Processes with Stationary Average Flow Speed

**Figure 3:**
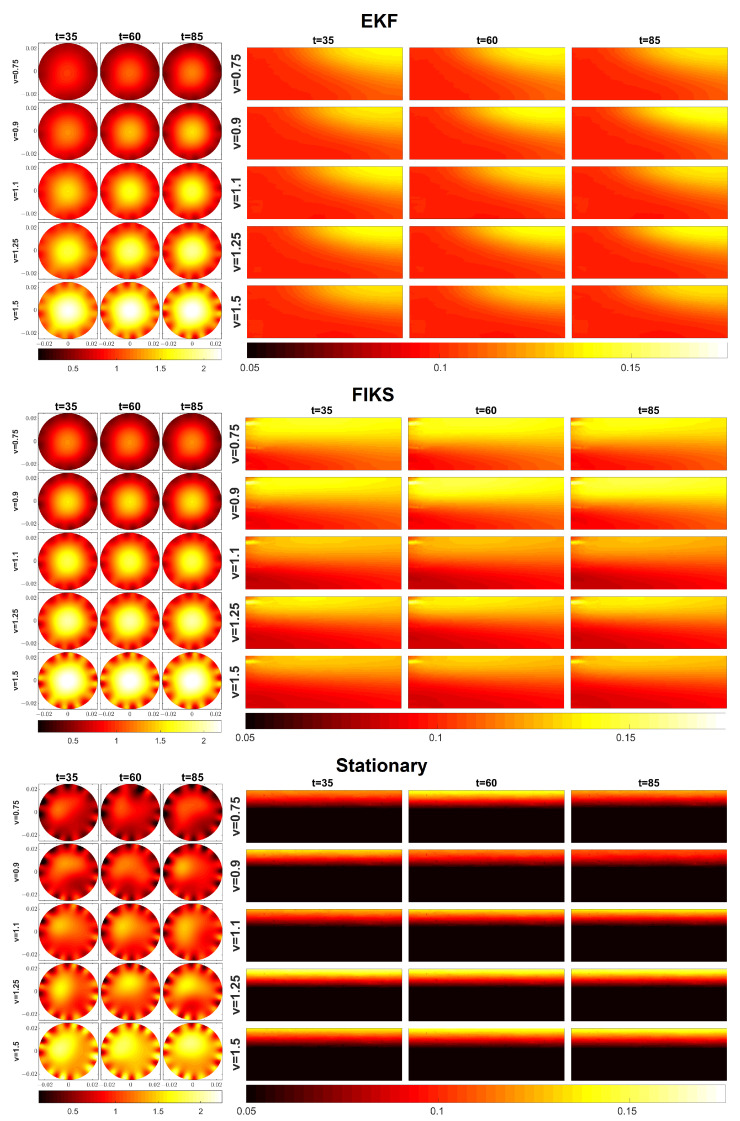
Cases 1–5: Reconstructions of the velocity fields (three leftmost columns) and oil fraction distributions (three rightmost columns) corresponding to three instants of time. The EKF, FIKS, and stationary reconstructions are shown on the top, middle, and bottom, respectively. The velocity fields are given in ${\mathrm{ms}}^{-1}$.

Figure 3 shows a comparison of the EKF, FIKS, and the stationary reconstructions in Cases 1–5 in which the average oil fraction was 10%. For all five test cases, the reconstructions of both the velocity profiles and the phase fraction distributions of oil are depicted corresponding to three instants of time.

Qualitatively, the velocity reconstructions given by EKF (Figure 3, the set of images in the top left corner) were rather feasible, except for inhomogeneities that emerged near the surface of the pipeline. These were most probably imaging artifacts caused by modeling errors related to the EMFT electrodes. Nevertheless, towards the center, the velocity profiles were somewhat symmetric with respect to the central axis of the pipeline, and the peak value was in the center in all EKF-based velocity reconstructions. At each flow case, the velocity profile did not vary much between the three time steps—this was a desired result, because the true velocity profile was expected to be constant in all these cases. As the average flow speed was increased from 0.75 ${\mathrm{ms}}^{-1}$ to 1.5 ${\mathrm{ms}}^{-1}$, the peak and average values in the velocity reconstructions increased correspondingly.

The EKF reconstructions for the phase fraction distributions of the oil are shown in top right corner of Figure 3. The phase fraction values were within the range of 0.09–0.15, which is reasonable as the true average phase fraction was 0.1 in Cases 1–5. Further, the phase fraction increased towards the top of the pipe, which was again reasonable because, at these flow speeds, oil and water are not perfectly mixed and oil, being the less dense component, tends to float on top of the water layers. However, in EKF reconstructions, the phase fractions were nearly uniform on the inflow boundary (left edge of the phase fraction images), and a higher oil content value on the top layers was reached only in the middle and end parts of the pipe segment. This is a known effect of EKF and was also shown in previous simulation studies [21], as well as when using EKF for a single modality ERT imaging [23,30,32]: The ET measurements were only sensitive to the electrical conductivity (and, thus, phase fraction) in the volume covered by the electrodes and not to conductivities near the ends of the pipe segment. Hence, when fluid entered the pipe segment through the inflow boundary, ET did not react to its variation from the background; it traced the variations only after it reached the vicinity of the first electrode layer. After the conductivity variation had been traced, the CD model carried the information on the transport of the fluid along the pipeline; for this reason, the inhomogeneous vertical profile of the phase fraction was visible on the outflow boundary, even though the measurements were not sensitive to the conductivity variations on the outflow boundary either.

The FIKS reconstructions for the velocity distributions were close to those given by the EKF; however, as expected, the variation between the velocity profiles at three instants of time was smaller than in the EKF. In the phase fraction reconstruction, the FIKS performed significantly better than the EKF: While in the EKF, the phase fraction profile was falsely uniform on the inflow boundary, the FIKS showed the inhomogeneity of the vertical profile along the length of the pipe segment. Apart from a small artifact in the top-left corner of the images, the phase fraction distributions looked feasible. In the FIKS reconstructions, the top layers of the pipeline showed a higher oil volume fraction. As mentioned above, this was the effect of oil being less dense than water. Furthermore, the FIKS reconstructions clearly showed the effect of the flow speed: While the contrast between the oil volume fraction in the top and bottom of the pipeline was high, the contrast became lower when the flow speed increased. This was a feasible result; indeed, the gradient of phase fraction decreased with the flow speed, because the higher fluid velocity induced stronger mixing of the phases.

The quality of the stationary reconstructions shown in Figure 3 is clearly poorer than the EKF and FIKS reconstructions: The velocity profiles were highly non-symmetric and contained even larger artifacts close to the electrodes than the EKF and FIKS estimates. Further, the oil volume fraction estimates had a very strong contrast between the top and bottom of the pipeline, but they did not feature the velocity-dependent mixing effect shown by the FIKS as discussed above.

The results of Cases 6–20 were similar to those of Cases 1–5 in Figure 3: In all cases, the FIKS gave qualitatively more feasible reconstructions than the EKF and the stationary approach. To assess the reconstructions quantitatively, we computed the spatial averages of the reconstructed velocities and compared those to the average flow speeds given by the ultrasound flow meter at each time instant. Table 2 shows the average relative errors of the velocity in all stationary flow cases. These were computed as

$${\mathrm{RE}}_{v}=\frac{1}{T}\cdot \sum_{t=1}^{T}\frac{|\frac{1}{A}\int_{S}{\hat{v}}_{z,t}dS-v_{z,t}^{\mathrm{US}}|}{v_{z,t}^{\mathrm{US}}}\cdot 100\%$$

where ${\hat{v}}_{z,t}$ is the posterior mean estimate of the flow speed, $v_{z,t}^{\mathrm{US}}$ is the average flow speed given by the ultrasound flow meter, and *A* is the cross-sectional area of the pipe. The average relative errors corresponding to all three estimates are listed in Table 2. In almost all test cases, ${\mathrm{RE}}_{v}$ of the FIKS reconstruction was significantly smaller than ${\mathrm{RE}}_{v}$ of the EKF and stationary reconstructions, as highlighted by the boldface numbers. On average, the ${\mathrm{RE}}_{v}$ was 3.79% for the EKF, 2.27% for the FIKS, and 3.54% for the stationary reconstruction.

In a respective manner, we compared the estimated average oil phase fractions based on the EKF, FIKS, and stationary reconstructions to the known average oil volume fractions, which were 0.1 in Cases 1–5, 0.2 in Cases 6–10, 0.3 in Cases 11–15, and 0.4 in Cases 16–20. The resulting average relative errors of the phase fraction, ${\mathrm{RE}}_{\phi }$, are listed in Table 3. Again, with boldface numerals emphasizing the results, the FIKS led to smaller values of ${\mathrm{RE}}_{\phi }$ than the EKF and stationary reconstructions in almost all test cases. Overall, the stationary reconstructions were much worse than the FIKS and EKF estimates, which supports the use of state estimation in dual-modal EMFT–ET imaging. To be more specific, the average values of ${\mathrm{RE}}_{\phi }$ for the EKF, FIKS, and the stationary reconstruction were 3.79%, 3.17%, and 7.52%, respectively.

Based on the results shown in Table 2 and Table 3, we concluded that the FIKS outperformed the EKF and stationary reconstructions in the conditions considered in the experiments. For this reason, the rest of this section focuses on the analysis of the FIKS-based estimates only.

**Figure 4:**
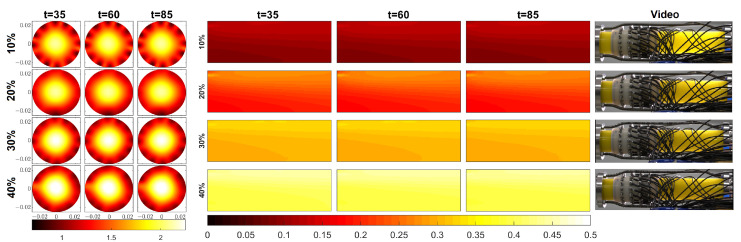
Cases 5, 10, 15, and 20, in which the true average velocity was 1.50 ${\mathrm{ms}}^{-1}$. FIKS-based reconstructions for the velocity field at three instants of time (three leftmost columns), along with the corresponding oil fraction distribution (Columns 4, 5, and 6) in cases where the true average oil fraction was 10%, 20%, 30%, and 40% (Lines 1, 2, 3, and 4, respectively). The velocity fields are given in ${\mathrm{ms}}^{-1}$. The rightmost column shows snapshots from the video in each case.

Figure 4 further illustrates the results of Cases 5, 10, 15, and 20, i.e., tests in which the true average velocity was 1.50 ${\mathrm{ms}}^{-1}$. Here, snapshots from the videos are shown side by side with the reconstructed oil volume fraction distributions in the respective test cases. As pointed out above, when the velocity of the fluid was high, the oil and water phases became mixed more thoroughly than in the cases with low velocities (cf. the FIKS reconstructions in Figure 3). Hence, also in Figure 4, the gradients in the oil volume fraction distributions are low. The values of these reconstructions, nearly homogeneous oil volume fraction distributions, were close to the true average values: 0.1, 0.2, 0.3, and 0.4 in Cases 5, 10, 15, and 20, respectively. The observation of oil and water being well mixed is also supported by the video snapshots shown on Figure 4: in each test case, the color of the mixture was rather uniform, but the differences between Cases 5, 10, 15. and 20 were clear; in Case 5, where the oil content was the smallest, the color of the fluid was light yellow, and in Case 20, corresponding to the highest oil content, the fluid color was the darkest (the rightmost column of Figure 4).

### 3.2. Case 21: Imaging of Process with Non-Stationary Average Flow Speed

**Figure 5:**
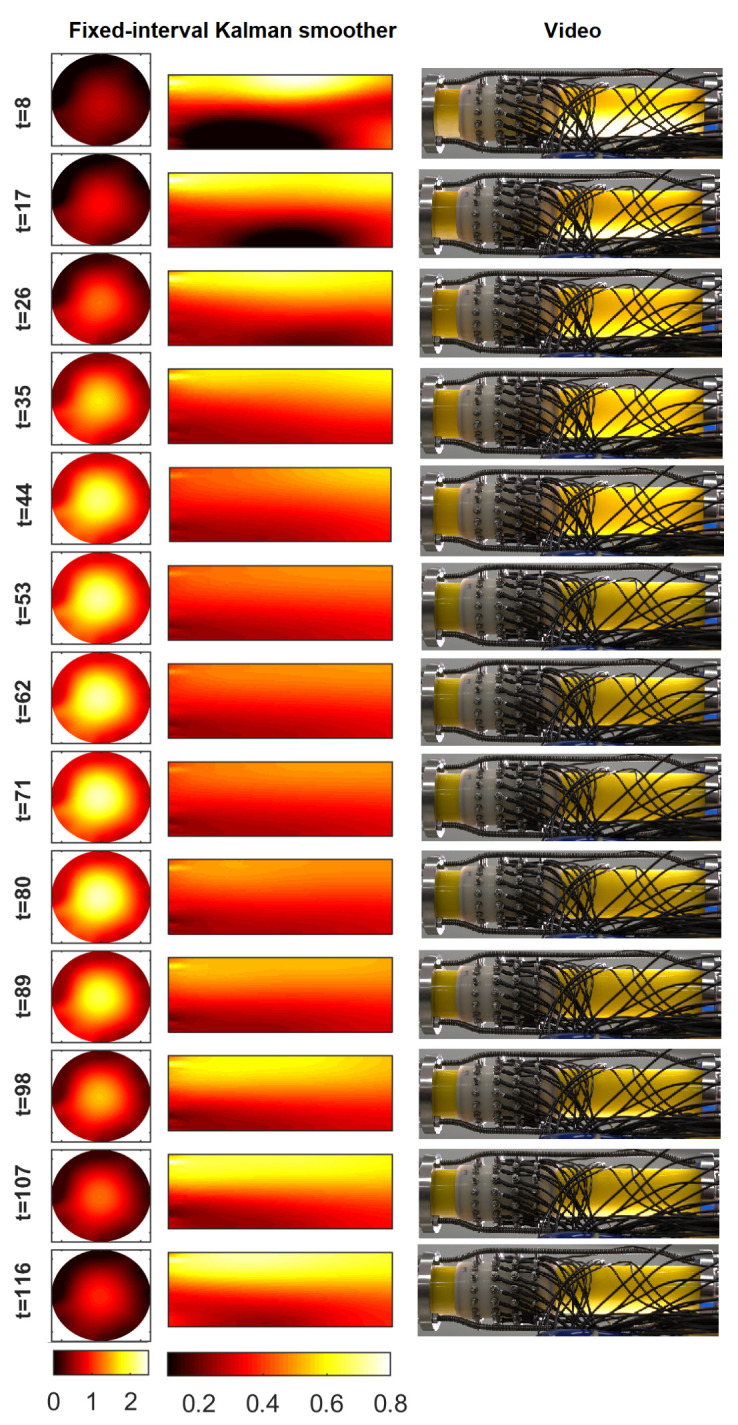
Case 21: FIKS-based reconstructions for the velocity field (left column) and oil fraction distribution (middle column) at 13 instants of time and snapshots from the video at the respective times. The velocity fields are given in ${\mathrm{ms}}^{-1}$.

Figure 5 illustrates the results of Case 21 in which the flow speed was non-stationary. The series of images shows the velocity profile and the oil volume fraction side by side with snapshots from the video. Again, the FIKS reconstructions were in agreement with the visual observation of the video. When the flow speed was low (t≤35), the oil volume fraction reconstructions showed clearly the separation of oil and water: oil accumulated at the top part of the pipe, and water was at the bottom. This was an expected result because the experiment started in the stratified flow regime, as also shown by the video snapshots from the beginning of the experiment. As the flow speed increased, the oil became more evenly distributed. In the time interval 53<t<80, the phases were rather well mixed, albeit the oil content was still larger on top than at the bottom of the pipe. Again, the average oil volume fraction was around 0.4, which equals the true average in Case 21. After t=89, when the flow speed decreased, the two phases were again separated, as shown by the volume fraction reconstructions.

The FIKS reconstructions in Figure 5 show a nearly stratified velocity field when the average velocity was low, i.e., at t≤35 and t≥89. In those periods of time, the flow speed was close to zero at the top of the pipe where the oil accumulated. This was an expected result: the flow of oil is slower than the flow of water, because oil has a higher viscosity than water. These findings align with the previous experimental work in [16]. Further, when the flow speed became higher (t≥35), the flow profile changed from nearly stratified to an axisymmetric profile, as shown by the velocity reconstructions.

**Figure 6:**
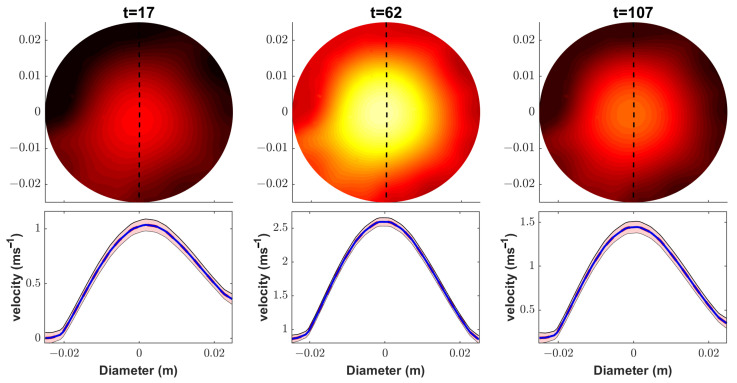
Case 21. The FIKS estimates for the velocity field at three instants of time (top row) and their vertical profiles at the respective times (bottom row). The position of the profile is marked in the 2D images with dashed black lines. In the 1D profiles, the conditional mean (CM) estimates are drawn with blue lines and the approximate 95% posterior credible intervals are marked with light red areas.

**Figure 7:**
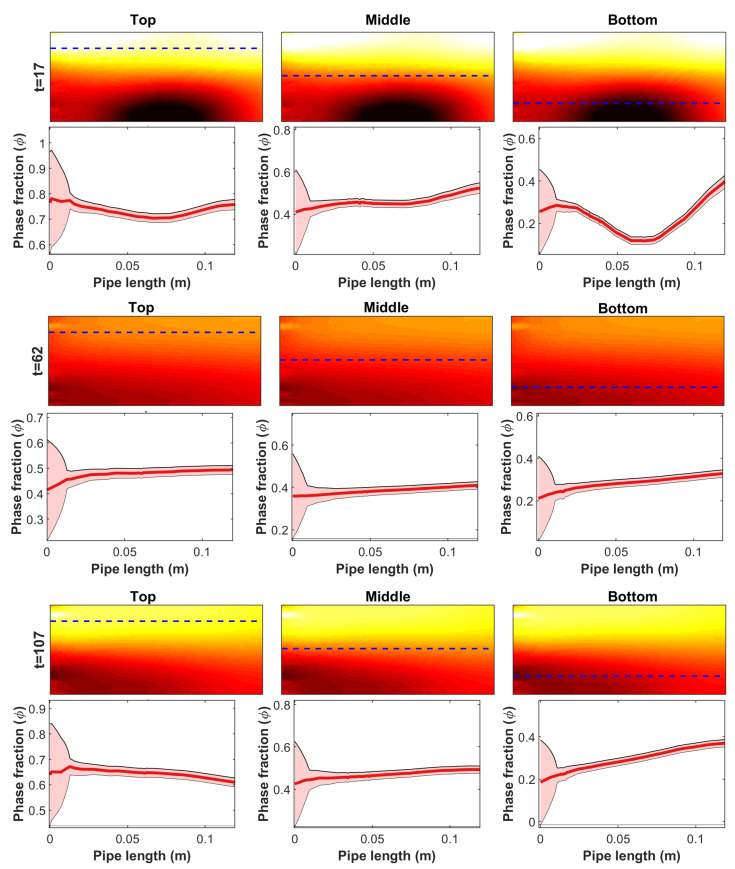
Case 21. The FIKS estimates for the oil fraction at three instants of time (Rows 1, 3, and 5) and their horizontal profiles (Rows 2, 4, and 6) from three heights (Columns 1–3). The heights of the horizontal profiles are marked in the 2D images with dashed blue lines. In the 1D profiles, the conditional mean (CM) estimates are drawn with red lines and the approximate 95% posterior credible intervals are marked with light red areas.

Figure 6 and Figure 7 illustrate the posterior uncertainties of the velocity field and the oil volume fraction, respectively. In these figures, the FIKS-based, approximately 95% posterior credible intervals (CI) of these quantities are plotted together with the respective conditional mean estimates at three instants of time. For easier interpretation, these estimates are plotted in 1D—along a vertical line from the middle of the pipe cross-section for the velocity and along horizontal lines at three heights for the oil volume fraction.

The narrow 95% CI observed in Figure 6 suggests that Bayesian smoothness estimation was feasible for accurately determining the uncertainties in the velocity field estimates at all time points. The 95% CI plots of the oil volume fraction (Figure 7) show an interesting phenomenon in the neighborhood of the inflow boundary: At all heights and all time instants, the CIs widen towards this boundary. This uncertainty was caused by the lack of sensitivity of the ET measurements to the volume fraction/conductivity outside the area covered by the electrodes, i.e., the same effect as described above in the context of EKF reconstructions; although the FIKS managed to enhance the volume fraction estimate near the inflow boundary by back-propagating the information of the future data, the estimate still remained more uncertain than in other parts of the pipe segment.

**Figure 8:**
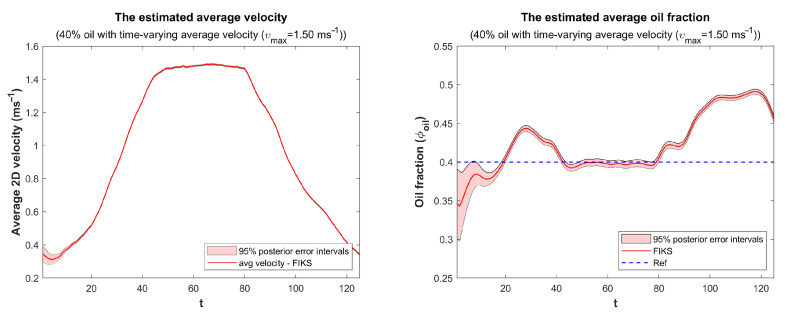
Case 21: Spatially averaged FIKS-based reconstructions of the velocity field (**left**) and oil fraction (**right**) as functions of time. The conditional mean (CM) estimates are drawn with red lines, and the approximate 95% posterior credible intervals are marked with light red color. The true average of the oil fraction, 0.4, is marked with a dashed blue line.

Finally, the spatially averaged FIKS-based reconstructions of the velocity field and oil fraction are plotted as functions of time in Figure 8. Again, the 95% CIs are plotted together with conditional mean estimates. The average velocity estimate was quite feasible; at the beginning of the experiment, it showed an average velocity somewhat above the true value (0.20 ${\mathrm{ms}}^{-1}$); after, this it decreased a bit, but after a short initialization period, the FIKS traced the increasing trend of the true velocity and followed the increase up to about a 1.50 ${\mathrm{ms}}^{-1}$ flow speed, where it remained from t=40 to t=80, after which it started decreasing. The CIs of the average velocity were very narrow, which implied a high certainty of the estimated average velocity values. It is worth to notice, however, that in this work, we did not account for all possible sources of modeling errors (such as those caused by unknown electrode contact impedances, imperfections in the generated magnetic fields, and discretization errors); for this reason, the CIs were probably unrealistically narrow. Furthermore, it is worth noting that the effectiveness of the velocity curve was further supported by its alignment with the measurements obtained from the commercial ultrasound flow meter.

During an initial period (t<20), the estimated average oil volume fraction was lower than the true value (0.40), but the 95% CI showed a large uncertainty for it. The oil volume fraction increased to about 0.45 before it stabilized to 0.40 for the duration of the high flow speed (40<t<80). When the flow speed decreased, the estimated oil volume fraction was again higher than the true average value. It is possible that the observed variation was not caused by an estimation error, but the average oil volume fraction actually varied in the imaged pipe section during the experiment. Indeed, when the flow speed was low, a large portion of the oil, being the lighter component, may have accumulate on top of the inclined pipe and vertical sections of the flow loop as at the beginning and at the end of the experiment. When the flow speed was high, the mixing of the phases made the fluids more evenly distributed also across the flow loop. This conclusion is supported by the fact that at high flow speeds, the estimated average oil fraction was very close to the true value.

### 3.3. Volumetric Flow Rate Estimates

To assess the feasibility of the volumetric flow rate estimates, we calculated references for the flow rates approximating those in the stationary flow tests (Cases 1–20); oil and water were evenly distributed along the length of the flow loop, and the steady-state flow was achieved. We note, however, that referring to the discussion in the above section, these approximations may not be accurate for the lowest flow speeds. Both these “theoretical” values of the volumetric flow rates and the dual-modal tomography-based FIKS estimates for the flow rates were computed using Equations (29) and (30). The relative errors of the FIKS estimates for the volumetric flow rates (i.e., relative errors with respect to the aforementioned theoretical values) are shown in Table 4. On average, the relative errors of the average volumetric flow rates of oil and water were 3.77% and 3.06%.

**Figure 9:**
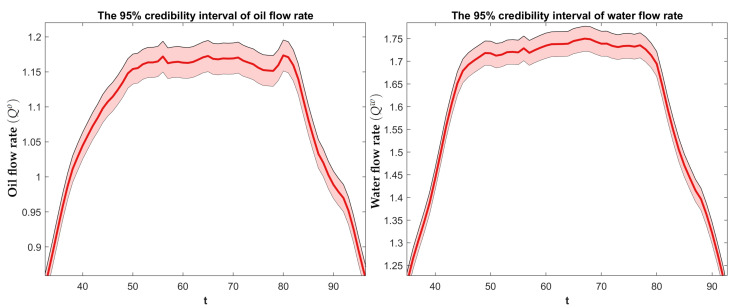
Case 21. The FIKS-based estimates for the oil (**left**) and water (**right**) flow rates as functions of time. The conditional mean (CM) estimates are drawn with red lines, and the approximate 95% posterior credible intervals are marked with light red areas.

The dual-modal tomography-based FIKS estimates for the volumetric flow rates of oil and water in the non-stationary test (Case 21) are plotted in Figure 9. Furthermore, the 95% CIs are depicted. The plots of both flow rates are quite symmetric with respect to time, showing the steady increase of the flow rates at the beginning of the experiment, the period of high flow rate in the middle of the experiment, and the steady decrease of the flow rate towards the end. Furthermore, the CIs seemed realistic, showing about ±5% uncertainty in the estimated values, which is in accordance with the range of relative errors given for the stationary estimates in Table 4. To further assess the feasibility of the flow rate estimates in the future, a separator tank for quantifying the true volumetric flow rates will be needed.

## 4. Conclusions

This experimental study investigated the feasibility of using the state estimation approach for dynamic image reconstruction in dual-modal EMFT-ET imaging of two-phase oil–water flow. By accounting for the interactions between the velocity field and phase fraction distributions, we estimated the spatio-temporally distributed velocity field and oil phase fraction using the extended Kalman filter (EKF) and fixed-interval Kalman smoother (FIKS). These quantities were further used to estimate the volumetric flow rates of the phases. Our experiments in a laboratory setup demonstrated that the state estimation approach was feasible, particularly when using the FIKS. The FIKS reconstructions accurately captured the dynamic changes of the non-stationary oil–water mixture. Moreover, the estimated quantities with FIKS were in good agreement with the average flow speed given by the ultrasound flow meter and the known average phase fraction of oil. Based on the reconstructed oil phase fraction and velocity field, the flow rate estimates were also accurate, with an average relative error of 3.77% for the oil volumetric flow rate and 3.06% for the water volumetric flow rate in the case of stationary flow speed. For non-stationary flow speed, the FIKS-based uncertainty estimates showed the accuracy of the approach, which was further visually confirmed by comparing the FIKS reconstructions to the snapshot of the experimental video.

In summary, our results demonstrated that the state estimation approach improved the dual-modal EMFT-ET imaging of the two-phase flow, particularly when compared to stationary imaging methods that neglect the contribution of velocity to the temporal evolution of phase fraction. By accounting for these interactions, the proposed approach has the potential to enhance the reliability and precision of dual-modal tomography, thereby enabling more accurate flow measurements. To further expand the potential of this approach, future research could investigate its applicability to more complex systems, such as fractured porous media, in order to assess its potential advantages and limitations. In the end, our proposed approach provides a promising tool for studying multi-phase flows, and offers a pathway towards broader applications in the field.

## Figures and Tables

**Table 1:** The test cases with different oil fractions and different flow speeds.

	Average Flow Speed (m/s)	0.75	0.90	1.10	1.25	1.50	Time- Varying
Oil Volume Fraction							
10%		Case 1	Case 2	Case 3	Case 4	Case 5	-
20%		Case 6	Case 7	Case 8	Case 9	Case 10	-
30%		Case 11	Case 12	Case 13	Case 14	Case 15	-
40%		Case 16	Case 17	Case 18	Case 19	Case 20	Case 21

**Table 2:** Cases 1–20: average relative errors (${\mathrm{RE}}_{v}$) of the average velocities for the EKF, FIKS, and stationary reconstructions.

EKF							FIKS					Stationary Approach				
	Average Flow Speed (m/s)	0.75	0.90	1.10	1.25	1.50	0.75	0.90	1.10	1.25	1.50	0.75	0.90	1.10	1.25	1.50
Oil Volume Fraction																
10%		4.04	4.36	2.45	3.22	2.60	**1.81**	**1.52**	**0.89**	**1.89**	**1.75**	4.79	1.99	1.37	2.14	3.64
20%		7.17	4.17	2.45	3.92	3.31	**3.97**	**3.03**	**1.07**	**2.62**	**2.97**	4.64	3.30	1.35	4.82	3.18
30%		3.40	6.45	3.54	4.76	3.24	**0.51**	4.14	1.97	**3.18**	**2.38**	7.36	**2.90**	**1.43**	4.25	3.16
40%		2.34	3.25	2.44	4.56	4.12	**0.47**	2.20	**1.19**	**3.97**	**3.86**	1.92	**1.60**	3.86	6.52	6.63

**Table 3:** Cases 1–20: average relative errors (${\mathrm{RE}}_{\phi }$) of the average oil volume fractions for the EKF, FIKS, and stationary reconstructions.

EKF							FIKS					Stationary Approach				
	Average Flow Speed (m/s)	0.75	0.90	1.10	1.25	1.50	0.75	0.90	1.10	1.25	1.50	0.75	0.90	1.10	1.25	1.50
Oil Volume Fraction																
10%		7.38	6.88	4.39	4.00	**4.14**	**6.12**	**5.05**	**2.83**	**2.43**	4.34	9.59	7.98	7.19	6.03	8.17
20%		4.32	6.07	**3.83**	3.86	**2.87**	**3.06**	**4.63**	4.59	**3.12**	2.92	7.96	8.10	6.32	6.42	7.53
30%		0.84	**1.86**	2.54	**2.28**	1.88	**0.45**	2.15	**2.02**	2.41	**1.60**	7.52	8.65	7.47	6.64	7.75
40%		5.69	5.44	**2.44**	**2.47**	2.57	**5.07**	**3.11**	2.69	2.55	**2.29**	7.31	6.72	7.93	7.11	7.91

**Table 4:** The average relative errors (%) of oil flow rate ($Q^{o}$) and water flow rate ($Q^{w}$).

Relative Errors (%) of $Q^{o}$							Relative Errors (%) of $Q^{w}$				
	Average Flow Speed (m/s)	0.75	0.90	1.10	1.25	1.50	0.75	0.90	1.10	1.25	1.50
Oil Volume Fraction											
10%		8.27	5.12	2.61	3.06	1.40	2.81	2.22	1.26	2.17	1.97
20%		6.57	5.28	2.16	3.75	3.22	3.92	3.69	1.77	2.83	3.15
30%		0.61	4.70	2.74	3.50	2.55	0.92	4.46	2.77	3.88	2.84
40%		3.31	5.78	2.91	2.71	5.14	4.13	3.25	2.62	6.41	4.22

## Data Availability

The data presented in this study are available upon reasonable request from the authors.
